# Aggressive atypical cystic fibromatosis of the pancreas: a case report and literature review

**DOI:** 10.3389/fonc.2025.1596535

**Published:** 2025-10-31

**Authors:** Xianhong Yang, Zhenji Xu, Yangyingqiu Liu, Tao Feng

**Affiliations:** ^1^ Department of Radiology, Zibo Central Hospital, Zibo, China; ^2^ Department of Education and Training, Zibo Central Hospital, Zibo, China; ^3^ Department of Thoracic Surgery, Zibo Central Hospital, Zibo, China

**Keywords:** aggressive fibromatosis, case report, desmoid tumor, pancreatic tumor, β-catenin

## Abstract

Aggressive fibromatosis of the pancreas (AFP) is a rare, non-epithelial, aggressive, benign soft tissue tumor of the pancreas that is characterized by β-catenin-positive expression. Due to its rarity, it is often misdiagnosed as a malignant pancreatic tumor. In this report, we present a case of aggressive atypical cystic fibromatosis that is specifically localized to the pancreas. We emphasize the key imaging features essential for diagnosing and assessing the aggressiveness of this condition, along with its pathological characteristics, pathogenesis, and surgical treatment. Progressive delayed enhancement, marked restricted diffusion, and aggressive features can aid in differentiation, and nuclear β-catenin positivity is highly characteristic of this condition. A 22-year-old woman was admitted following the incidental discovery of a cystic-solid pancreatic mass during a routine medical examination. This patient underwent preoperative ultrasound, computed tomography, and magnetic resonance imaging. The identified lesion was subsequently surgically resected, and a histopathological examination confirmed the presence of AFP. Notably, the tumor was found incidentally, as the patient was asymptomatic. Radiologists should consider the possibility of aggressive fibromatosis in patients presenting with pancreatic tumors, as surgical resection can lead to a cure. Therefore, comprehensive imaging analysis is crucial for making an accurate preoperative diagnosis and providing appropriate treatment.

## Introduction

Desmoid tumors—also referred to as aggressive fibromatosis (AF)—are rare, particularly when they develop in the intra-abdominal region. They typically occur in the pelvic cavity and mesentery (1), and their occurrence in the pancreas is even rarer, with approximately 32 cases reported in the literature (**Table 1**) (1–26). Aggressive fibromatosis of the pancreas (AFP) is a benign soft tissue tumor that displays aggressive behavior (27). A definitive diagnosis of AF is established through pathology and immunohistochemistry, specifically by detecting β-catenin-positive expression. Additionally, imaging plays a crucial role in the diagnostic process. Progressive delayed enhancement, marked restricted diffusion, and aggressive features can aid in differentiation. This study presents a case of atypical cystic AFP. The correlation between the imaging findings, the surgical results, and the pathological characteristics was analyzed comprehensively.

**Table 1:** Summary of the previous case reports of aggressive fibromatosis of the pancreas and the present case.

Case	Age	Sex	Location	Size (cm)	Imaging presentation	β-catenin IHC	Ki67 index (%)	Management	Follow-up (months)	Outcome
Present case	22	F	Tail	5.0	Solid cystic	Positivity	10%	Resection	22	No recurrence
1 (1)	20	F	Tail	6.6	Solid cystic	N/A	N/A	Resection	108	No recurrence
2 (1)	25	F	Body	4.8	Solid	Positivity	N/A	Biopsied	N/A	Unknown
3 (1)	33	F	Body	5.8	Solid	N/A	N/A	Biopsied, sorafenib, observed tumor without resection	30	Tumor stable
4 (1)	54	F	Body	2.7	Solid cystic	Positivity	N/A	Resection	N/A	Unknown
5 (1)	67	F	Body	3.3	Solid	Positivity	N/A	Resection	6	No recurrence
6 (1)	74	M	Body	2.4	Solid	Positivity	N/A	Biopsied, observed tumor without resection	30	Tumor stable
7 (1)	78	F	Body	2.4	Solid	Positivity	N/A	Biopsied, observed tumor without resection	6	Tumor stable
8 (2)	42	M	Body and tail	26.0	Solid	Positivity	N/A	Resection	60	No recurrence
9 (3)	21	F	Head and body	6.0	Solid	N/A	1%	Resection	N/A	Death
10 (4)	38	M	Body	5.0	Solid	N/A	N/A	Resection	24	No recurrence
11 (5)	15	F	Body and tail	36.0	Solid	Positivity	N/A	Resection	12	No recurrence
12 (6)	20	F	Body	7.5	Solid	Positivity	N/A	Resection	15	No recurrence
13 (7)	63	F	Tail	5.1	Solid	Positivity	N/A	Resection	N/A	No recurrence
14 (8)	60	M	Tail	3.0	Solid	Positivity	N/A	Resection	36	No recurrence
15 (9)	54	F	Head	5.2	Solid	N/A	N/A	Resection	N/A	No recurrence
16 (10)	41	M	Head	1.9	Solid cystic	Positivity	N/A	Resection	24	No recurrence
17 (11)	45	M	Body	12.0	Solid cystic	Positivity	N/A	Resection, NSAIDs, and sorafenib	12	Recurrence at 12 months
18 (12)	26	M	Uncinate process	4.5	Solid	Positivity	1%	Resection	40	No recurrence
19 (13)	41	M	Body and tail	40.0	Solid	Positivity	N/A	Resection	12	No recurrence
20 (14)	49	M	Diffuse	4.3	Solid	Positivity	N/A	Resection	N/A	Unknown
21 (15)	23	F	Body and tail	10.0	Solid	Positivity	N/A	Resection	36	No recurrence
22 (16)	17	M	Body	4.2	Solid cystic	Positivity	N/A	Resection, sulindac, tamoxifen, systemic chemotherapy for recurrence	24	Recurrence at 9 months
23 (17)	68	M	Tail	5.0	Solid cystic	Positivity	2%	Resection	60	No recurrence
24 (18)	66	M	Body	3.0	Cystic	Positivity	N/A	Resection	8	No recurrence
25 (19)	13	M	Tail	10.0	Cystic	Positivity	N/A	Resection	19	No recurrence
26 (20)	15	M	Body and tail	20.0	Cystic	Positivity	N/A	Resection	2	No recurrence
27 (21)	75	F	Body	8.0	Solid	Positivity	N/A	Resection	N/A	Unknown
28 (22)	57	F	Head	10.0	Solid	Positivity	N/A	Biopsy, celecoxib	18	No recurrence
29 (23)	63	M	Tail	6.5	Solid	Positivity	N/A	Resection	9	No recurrence
30 (24)	17	M	Neck and body	8.6	Solid cystic	N/A	N/A	Resection	40	No recurrence
31 (25)	17	M	Tail	N/A	Cystic	N/A	N/A	Resection	6	No recurrence
32 (26)	11	M	Tail	10	Solid cystic	Positivity	2%	Resection	10	No recurrence

IHC, immunohistochemical.

## Case report

**Figure 1:**
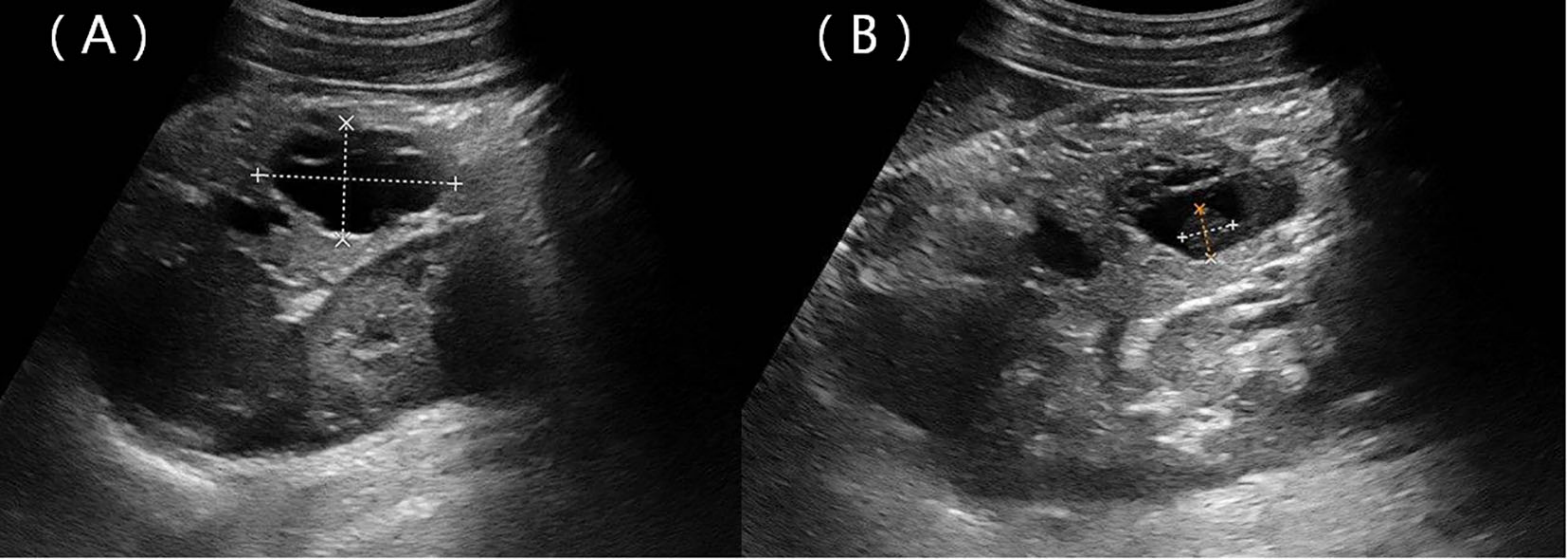
Ultrasound showing a hypoechoic mass located in the body and tail of the pancreas with clear margins **(A)** and a nodule with strong echoes at the edge **(B)**.

**Figure 2:**
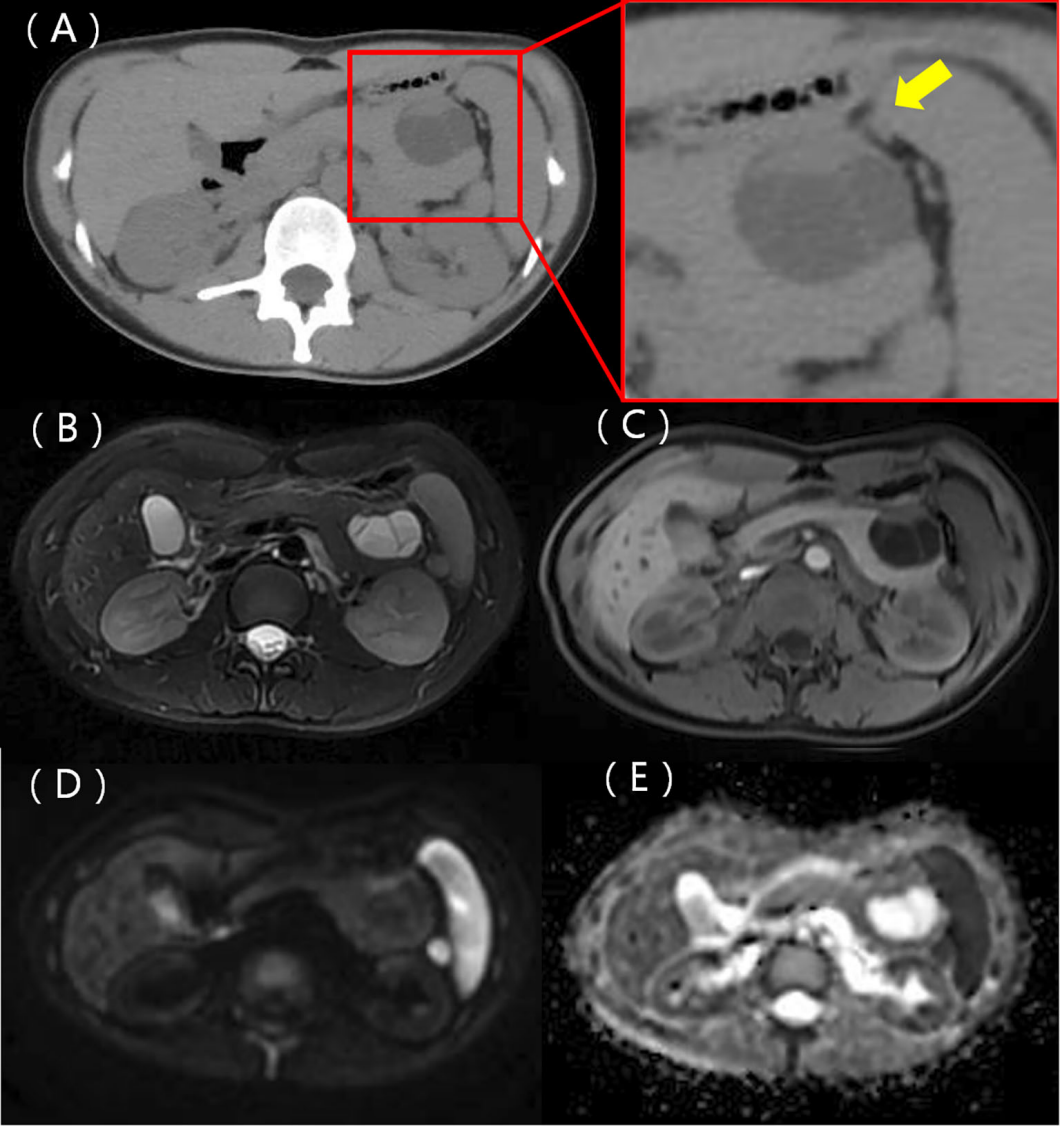
A CT scan identified a cystic-solid mass in the body and tail of the pancreas. The mass exhibited a regular shape, with solid components having equal density and septa, not clearly demarcated from the stomach (represented by the yellow arrow) **(A)**. MRI T1WI shows solid components, with the septa appearing isointense, while the cystic components were hypointense **(B)**. T2WI illustrates lightly hyperintense solid components and septa with hyperintense cystic components **(C)**. A diffusion-weighted image depicts the solid components and septa as hyperintense **(D)** and hypointense on the ADC map **(E)**.

**Figure 3:**
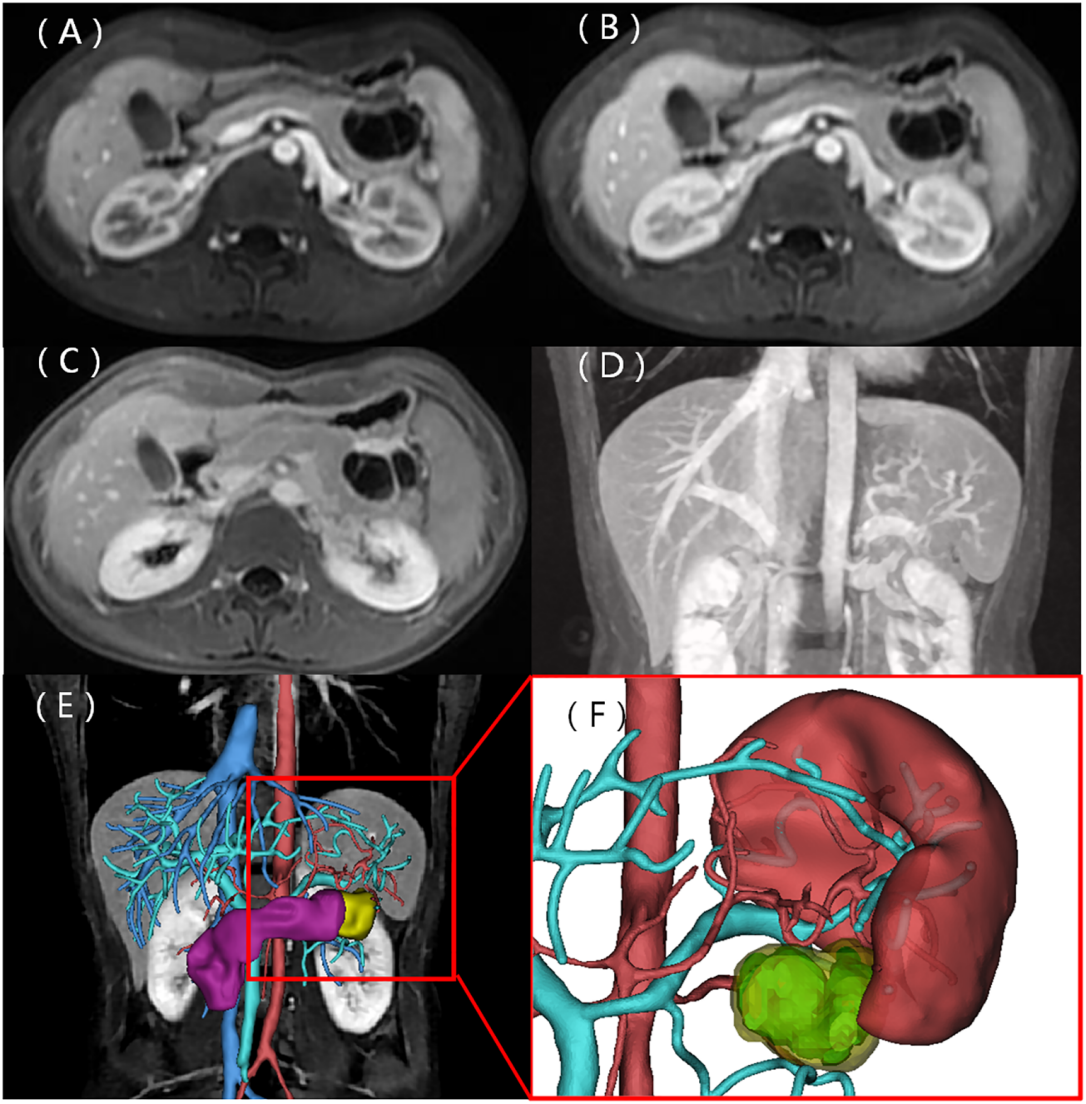
Contrast-enhanced MRI scans. The solid components and septa were slightly enhanced in the arterial phase, with delayed enhancement observed in the venous and delayed phases **(A–C)**. The coronal MIP image displays enlargement of the portal vein and tortuous dilation of blood vessels in the splenic hilum **(D)**. **(E)** Preoperative three-dimensional MRI of the tumor and adjacent structures. **(F)** Cystic (light blue) and solid (yellow) components of the tumor.

A 22-year-old woman was admitted to the hospital in August 2022 after a routine medical checkup revealed a cystic-solid mass in her pancreas. The patient’s past medical history, personal history, and family history were unremarkable. Physical examination of the abdomen revealed no notable findings. An ultrasound revealed a cystic mass in the tail of the pancreas with clear margins, multiple hyperechoic septa, and isoechoic nodules at the edge (**Figure 1**). Furthermore, a computed tomography (CT) plain scan revealed a cystic-solid mass in the body and tail of the pancreas, which appeared regularly shaped with solid components of equal density and septa (**Figure 2A**), measuring approximately 3.6 × 2.5 cm. There was no clear demarcation of the lesion from the adjacent organs and the peritoneum. To further assess the nature of the lesion, the patient underwent magnetic resonance imaging (MRI). The MRI findings revealed a cystic-solid mass in the body and tail of the pancreas with septa. The solid components and septations appeared isointense on the T1-weighted image (T1WI), while they were slightly hyperintense on the T2-weighted image (T2WI). The cystic components were hypointense on T1WI and hyperintense on T2WI (**Figures 2B, C**). The lesion was found to adhere tightly to the adjacent organs and the peritoneum. On diffusion-weighted imaging (DWI), the solid components displayed hyperintensity (*b* = 800 s/mm^2^) and hypointensity on the apparent diffusion coefficient (ADC) map (**Figures 2D, E**). Gadolinium-enhanced scanning revealed a slight enhancement of the solid components and septa in the arterial phase (**Figure 3A**), followed by continuous enhancement in the venous and delayed phases (**Figures 3B, C**). However, the cystic component did not display enhancement. The maximum intensity projection (MIP) image indicated an enlargement of the portal vein and tortuous dilation of blood vessels in the splenic hilum (**Figure 3D**). Moreover, **Figures 3E, F** display the preoperative three-dimensional MRI of the tumor and adjacent structures. The preoperative diagnosis was a solid pseudopapillary tumor of the pancreas.

**Figure 4:**
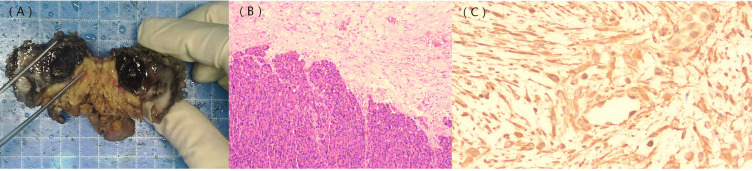
Gross pathology image; histological and immunohistochemical findings. The gross pathology image shows a solid cystic lesion **(A)**. Hematoxylin–eosin staining indicates densely arranged tumor cells and normal pancreatic tissue **(B)**. β-catenin staining shows positive signals in the nuclei of the tumor cells [**(C)**, ×400].

The patient underwent a surgical operation after general anesthesia. During the operation, a tumor of approximately 5.0 cm in diameter was found in the pancreatic body near the tail. The tumor tightly adhered to the stomach and jejunum. The pancreatic and splenic vessels were carefully separated along Toldt’s gap, revealing that the tumor was firmly attached to the retroperitoneum, posterior to the stomach, and adjacent to the beginning of the jejunum. Additionally, the splenic vein was encased by the tumor, leading to regional portal hypertension and localized varicose veins. Following surgery, a histopathological examination confirmed the diagnosis of pancreatic aggressive atypical cystic fibromatosis. Immunohistochemical staining was positive for β-catenin (**Figure 4**), vimentin, P53, and Ki-67 (10%) and negative for CKAE1/AE3,CK8/18, CD56, CK7, CK19, Syn, CgA, NSE, ER, PR, α-Inhibin, M C5AC, MUC2, MUC6, CDX2, Dog-1, CD117, CD34, SMA, desmin, HMB-45, Melan-A, and S-100. The tumor exhibited a low mitotic count (​​1 to 2/10 high-power fields ​​) and a stromal composition characterized by a ​​collagen proportion of 20%.

The patient underwent surgery without further treatment and remained disease-free for 22 months afterward. There were no adverse or unexpected events, and the clinician assessed a good prognosis. The patient felt fortunate to have undergone surgery and achieved a cure.

## Discussion

AF of the pancreas is a rare, non-epithelial, aggressive, benign soft tissue tumor. It commonly occurs in individuals aged 25 to 35, and it is more prevalent in women (27). While this tumor does not possess the ability to metastasize, it can invade neighboring structures. Its imaging characteristics depend on the degree of fibroblast proliferation, the relative proportion of collagen fibers, and the vascular supply of the entire tumor. The compact arrangement of fibrous components within the lesion can limit the diffusion of water molecules, leading to a high signal intensity on DWI. Additionally, the fibrous components within tumors can continuously accumulate contrast agents, resulting in a progressive enhancement pattern (28). In this case, the tumor demonstrated hyperintensity on DWI, along with noticeable delayed gadolinium enhancement.

The imaging presentation of AFP requires differentiation from other pancreatic malignant tumors and cystic pancreatic lesions with overlapping features. Compared with pancreatic ductal adenocarcinoma (PDAC), while both may exhibit ill-defined margins and vascular encasement, PDAC predominantly affects older adults with a peak incidence at 60–80 years (29) and typically causes pancreatic duct dilation (30, 31). Crucially, PDAC demonstrates metastatic potential (32), whereas fibromatosis remains locally aggressive. Intraductal papillary mucinous neoplasm (IPMN) characteristically communicates with the pancreatic duct, causing ductal dilatation and often showing vividly enhancing mural nodules (33). The solid component of a pancreatic neuroendocrine tumor shows obvious enhancement in the early phase after contrast administration (34). Pancreatic mucinous cystic neoplasms exhibit expansive growth and may demonstrate eggshell calcification (35). The initial misdiagnosis of our case as a solid pseudopapillary tumor (SPT) was primarily attributable to several factors. First, the demographic profile of AFP is highly consistent with SPT; both prefer young women and are frequently discovered incidentally (36). Second, a substantial overlap exists in the imaging characteristics between the two. When SPTs present as cystic-solid masses, their imaging characteristics—such as mixed components, internal septations, and progressive enhancement—closely resemble those observed in our case, creating a diagnostic challenge. Finally, the disparity in disease prevalence introduces a bias. AFP is exceptionally rare, with only sporadic cases reported in the literature. In contrast, although SPT itself is uncommon (36), it remains a more frequently encountered entity in the differential diagnosis of pancreatic masses in young women. Consequently, radiologists and surgeons are inclined to prioritize more probable diagnoses over exceedingly rare ones, leading to an anchoring bias toward SPT in this clinical scenario. Nevertheless, key distinguishing features exist between these two entities: SPT typically presents with a more well-defined capsule and more frequently exhibits hemorrhagic degeneration (36), whereas AFP tends toward invasive growth, often exhibiting fibrous adherence to surrounding structures rather than mere displacement.​ Although there was a discrepancy in the preoperative diagnosis, the surgical resection is the primary treatment for both SPT and AFP. However, by recognizing the possibility of this entity preoperatively, the radiologist can include it in the differential diagnosis. This would alert the surgeon to prepare for a more complex procedure, given that aggressive fibromatosis is often ill-defined and invasive, requiring a meticulous evaluation of its relationship with surrounding vessels and adjacent organs.

The definitive diagnosis of AFP depends primarily on pathology and immunohistochemistry. Histopathological examination revealed that the tumors consist of varying proportions of spindle-forming fibroblasts and collagen fibers. Additionally, a significant majority (90%–95%) of the tumors exhibited point mutations in the β-catenin (CTNNB1) gene, with the positive expression of β-catenin serving as the primary diagnostic criterion for pancreatic fibromatosis. CTNNB1 is involved in the formation of the Wnt/β-catenin signaling pathway, which promotes the transcription of pro-proliferative and anti-apoptotic genes, thereby driving uncontrolled cellular proliferation and tumorigenesis (37). Absence of staining for cytokeratins (CKAE1/AE3, CK8/18, CK7, and CK19) effectively excludes carcinoma (38), including primary PDAC and its variants. Similarly, negativity for neuroendocrine markers (Syn, CgA, and CD56) excludes a pancreatic neuroendocrine tumor (39).​​ CD34 negativity​​ helps exclude a ​​solitary fibrous tumor (40). In this case, immunohistochemistry revealed β-catenin-positive expression alongside the pathological characteristics observed under the microscope. Combined with the negative results mentioned above, these collectively led to the definitive diagnosis of AFP and excluded all other major differential diagnoses. This case demonstrated a Ki-67 proliferative index of approximately 10%, which is inconsistent with the low indices (typically 1% to 2%) most commonly reported in the literature for AFP (3, 12, 17, 32). This discrepancy may reflect the atypical nature of this particular case and highlights the potential biological heterogeneity of this disease. The local invasiveness observed in our patient, as evidenced by the invasion into surrounding structures as seen on imaging and during surgery, may be associated with this higher proliferative activity (41). While diagnostic confirmation remains rooted in classic histology and β-catenin immunophenotype, the Ki-67 index could potentially serve as a supplementary indicator of a more aggressive clinical subtype. Further accumulation of cases with detailed long-term follow-up is necessary to determine whether a higher Ki-67 index correlates with a greater risk of recurrence or a more aggressive clinical course in AFP. The tumor exhibited a ​​low mitotic count (1 to 2/10 high-power fields)​​, which is characteristic of its biologically ​​benign nature (42). ​​Semi-quantitative assessment​​ revealed a ​​collagen proportion of approximately 20%​​, indicating a ​​highly cellular lesion​​ with a relatively scant collagenous stroma (43). This finding suggests a proliferative cellular state, which is consistent with the tumor’s ​​elevated Ki-67 proliferation index.

The operative record documented an indistinct demarcation between the lesion and neighboring organs and peritoneum, indicating the invasiveness of the lesion. An enlarged portal vein and the tortuous dilation of blood vessels in the splenic hilum, observed in the MIP image, were also confirmed during surgery. The splenic vein was wrapped by the tumor, leading to these alterations. Despite its aggressive behavior, the tumor is usually benign in nature and can be cured via surgical resection. Therefore, clinicians should exercise caution to avoid misdiagnosis and unnecessary overtreatment.

The management paradigm for AF has substantially evolved in recent years toward a multidisciplinary and individualized strategy. ​​Initial active surveillance​​ is now recommended as the preferred approach for asymptomatic patients (44). For patients with ​​progressive or symptomatic disease, ​​systemic therapy​​ such as sorafenib or pazopanib has demonstrated significant clinical efficacy, establishing these agents as first-line options,: particularly for unresectable cases (45, 46). Radiotherapy (13) and interventional radiology (47) are emerging treatments. Nevertheless, ​​radical surgical resection​​ with R0 margins remains the cornerstone of treatment for localized, resectable lesions (1). In the present case, successful resection without adjuvant therapy was performed. The patient’s sustained disease-free status at the 22-month follow-up aligns with reported surgical outcomes, reinforcing resection as a definitive management strategy for appropriately selected patients with a localized disease. While the potential for local recurrence exists in AFP, the current literature is limited to only two reports (11, 16). Therefore, it is important to consider evidence from studies of AF at other sites. Studies have shown that specific patient characteristics and tumor features are closely associated with recurrence risk. —for instance, younger patients, tumors located in limb regions, and those exceeding 10 cm in size are all linked to higher recurrence rates (48). Recent studies recommend active surveillance over immediate surgical intervention for the majority of patients (49). For patients who opt for surgery, the risk of postoperative recurrence correlates closely with surgical margins. Research indicates that patients with microscopic or macroscopic residual tumors face a significantly higher risk of recurrence. Therefore, ensuring complete removal of surgical margins is crucial to reducing recurrence rates (50). In patient counseling, clinicians should develop personalized treatment and monitoring plans tailored to individual circumstances. For patients at high risk of recurrence, closer monitoring and more aggressive treatment strategies may be required. Additionally, patients should be informed about the potential risks and benefits of surgical procedures and other therapeutic options to facilitate informed decision-making. In conclusion, the management of hard fibroma requires a comprehensive consideration of the patient’s individual needs, tumor molecular characteristics, and treatment risk–benefit ratios. Future research should continue to explore more precise molecular diagnostic methods and personalized treatment plans to further improve the patients’ quality of life and treatment outcomes (51, 52).

The strength of this case report lies in its description of a rare situation that can potentially be encountered by any radiologist or general surgeon. However, there are some limitations, including the inherent limitations of a retrospective case record and the lack of comprehensive genetic testing.

In summary, AFP is a rare, nonepithelial, aggressive, benign soft tissue tumor of the pancreas. Preoperative imaging frequently cannot distinguish this entity from a malignant pancreatic tumor. It is crucial to understand the imaging and pathological characteristics for the purpose of diagnosing the disease accurately, which can facilitate correct preoperative assessment and ensure appropriate treatment.

## Data Availability

The original contributions presented in the study are included in the article/supplementary material. Further inquiries can be directed to the corresponding author.
